# Missing Data Imputation Method Combining Random Forest and Generative Adversarial Imputation Network

**DOI:** 10.3390/s24041112

**Published:** 2024-02-08

**Authors:** Hongsen Ou, Yunan Yao, Yi He

**Affiliations:** School of Naval Architecture, Ocean and Energy Power Engineering, Wuhan University of Technology, Wuhan 430063, China; wut_hongsen@163.com (H.O.); 261803@whut.edu.cn (Y.H.)

**Keywords:** random forest, generative adversarial interpolation network, time-series data, data interpolation

## Abstract

(1) Background: In order to solve the problem of missing time-series data due to the influence of the acquisition system or external factors, a missing time-series data interpolation method based on random forest and a generative adversarial interpolation network is proposed. (2) Methods: First, the position of the missing part of the data is calibrated, and the trained random forest algorithm is used for the first data interpolation. The output value of the random forest algorithm is used as the input value of the generative adversarial interpolation network, and the generative adversarial interpolation network is used to calibrate the position. The data are interpolated for the second time, and the advantages of the two algorithms are combined to make the interpolation result closer to the true value. (3) Results: The filling effect of the algorithm is tested on a certain bearing data set, and the root mean square error (RMSE) is used to evaluate the interpolation results. The results show that the RMSE of the interpolation results based on the random forest and generative adversarial interpolation network algorithms in the case of single-segment and multi-segment missing data is only 0.0157, 0.0386, and 0.0527, which is better than the random forest algorithm, generative adversarial interpolation network algorithm, and K-nearest neighbor algorithm. (4) Conclusions: The proposed algorithm performs well in each data set and provides a reference method in the field of data filling.

## 1. Introduction

With the development of science and technology, the world has entered the era of big data; equipment will generate massive time-series data during operation. Through data change analysis, equipment status changes can be evaluated and predicted, and equipment fault diagnosis and operation and maintenance support can be guided. However, due to the instability of sensing equipment or the influence of external factors, the collected time-series data may be incomplete, and the missing parts of the time-series data also contain data change patterns. If the missing parts are not dealt with, key information may be missed during data analysis, making it difficult for the established prediction model to extract accurate trend information from existing data, affecting the prediction accuracy of the prediction model [1]. Therefore, it is of great significance to fill in the missing parts of the data in order to restore the real data trends, accurately mine data patterns, and better guide subsequent reliability assessment work.

Traditional missing-data-filling methods are divided into two categories: deletion methods and filling methods [2]. The deletion method deletes samples with missing data, but it will further reduce the number of samples, causing some samples to be missing either in part or in full, which is not conducive to the prediction model mining the regular information hidden in the data. The filling method learns the non-missing data and trains the prediction model to find the potential relationship between the data, thereby generating data corresponding to the missing parts. Currently popular interpolation methods are statistical interpolation and machine learning interpolation. The statistical interpolation method mainly uses statistical knowledge to fill in data, such as the mean, median, mode, etc. However, this method is suitable for situations where there are less missing data and is only suitable for linear assumptions, unlike nonlinear data in reality. Most cases do not match. The data-filling method based on machine learning uses machine learning to fit the distribution of the training data set. For missing data, it uses the existing distribution function and combines the data characteristics around the missing value to automatically generate data for the corresponding missing position. For actual situations, the non-linear data-filling effect is better.

Compared with the classic statistical method for data filling, using machine learning algorithms to fill time-series data does not require prior knowledge and certain mathematical expressions. However, the data pattern of data sets with time-series properties is complex, and only simple machine learning algorithms are used. The imputation of missing data cannot meet accuracy requirements. In order to solve the problem that it is inconvenient to use traditional statistical filling methods to form definite mathematical expressions for time-series data in traffic flow, Wang et al. [3] regarded traffic flow data as a superposition of multiple simple modes and proposed a matrix decomposition method to extract data trend characteristics and then combine it with a tree neural network to obtain interpolation data, and finally integrate the interpolation results of multiple simple models to improve the interpolation accuracy of missing data. Xu et al. [4] used the isolated forest algorithm to test outliers on wind power output power data, marked the detected outliers as missing values, and used a multivariable time-series interpolation method based on a generative adversarial network to interpolate incomplete data. Ding [5] proposed an improved model, RVGAN-TL, that combines a genetic algorithm and transfer learning to solve the imbalanced learning problem of tabular data. Yin Hao [6] used a Wasserstein divergence generative adversarial network to learn the timing rules and coupling relationships of photovoltaic data, optimizing the input of the generator so that the generated samples are as close as possible to the true value. Marc Bresson [7] utilized GANs for domain transfer to convert multi-channel images from 3D scenes to realistic images. Chenchen Zhang [8] and others used a GAN for adversarial training that can generate missing traffic status data based on the known traffic status data of the road network. The missing data interpolation algorithm based on generative adversarial networks proposed by Lee et al. [9] is applied in the semiconductor industry. Experimental results show that the algorithm has excellent results when the missing data rate is less than 20%. Xu et al. [10] used random forest regression algorithm to evaluate and analyze groundwater quality. Zhang [11] mainly designed a cloud-based missing-data-filling method, which can integrate multiple different filling algorithms to process missing data simultaneously. Ioannis Papailiou [12] filled in missing values by comparing artificial neural networks based on multi-layer perceptrons and multiple linear regression. The results showed that the artificial neural network (ANN) training time is longer but the results are better than multiple linear regression. Thompson [13] proposed using the Wasserstein GAN algorithm to generate high-speed dynamic data. Dong et al. [14] applied the GAIN algorithm in research on filling missing clinical data. The work of Bernardini [15] and others is based on generative adversarial networks under clinical conditions, using nonlinear and multivariate information between data to fill missing values, which is more robust than other algorithms. Wang [16] and others proposed a spatiotemporal attention generative adversarial network algorithm (STA-GAN) to learn short-term correlation and dynamic spatial correlation in satellite data in order to improve the filling effect when the missing data rate is large. Zhang et al. [17] proposed a self-attention generative adversarial network filling model (SA-GAIN) to fill in missing data of traffic flow. Sahoo et al. [18] compared and verified the effectiveness of the K-nearest neighbor algorithm, self-organizing map algorithm, random forest algorithm, and feed-forward neural network algorithm in interpolating missing values in precipitation data. Qu et al. [19] used a multiple optimization data-filling method based on a generative adversarial network to fill in the missing values of the operating data of wind turbines, and proposed two optimization functions.

In terms of data filling, interpolation methods based on machine learning are widely used. Among them, the generative adversarial network performs better because it can fill data without prior knowledge and can generate new filled data samples by learning the distribution characteristics of existing data. However, it also faces training difficulties. When the amount of data is small, the training effect is not good, and there are shortcomings and challenges such as model collapse or lack of diversity. This study proposes a data-filling method based on random forests and generative adversarial interpolation networks to address the situation where the data collection effect may not be ideal under experimental conditions, resulting in missing data. Combined with the strong interpretability and applicability of random forests in dealing with nonlinear problems, a random forest is used to perform the first interpolation and filling of the data. In order to avoid unsatisfactory filling results at one time, the output value of the random forest algorithm is used as the input value of the generative adversarial interpolation network, and the generative adversarial interpolation network is used again for data filling. Through two-stage data interpolation, the advantages of the two algorithms are combined, and the output result is used as the final result of data filling to realize the interpolation of missing data.

## 2. Materials and Methods

**Figure 1:**
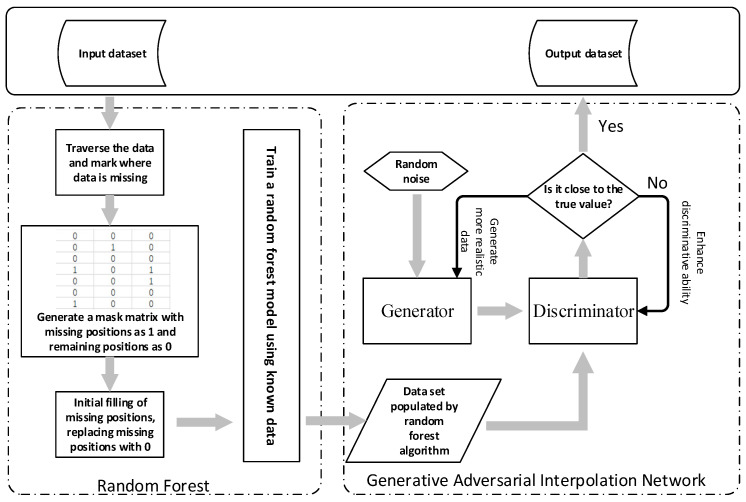
Program running framework.

Aimed at problems such as unsatisfactory filling results and large data errors when a single machine learning algorithm is applied to fill missing values in time-series data, an algorithm based on a random forest and a generative adversarial interpolation network is proposed, as shown in Figure 1.

Firstly, a random forest was used to perform distribution fitting and data filling on the data collected from the experiment. In order to avoid the problems of an insufficient filling effect and insufficient accuracy, a generative adversarial interpolation network was used to perform regression fitting on the filled data again. After the secondary fitting, we checked whether the error between the generated data and the real data reached an acceptable range.

### 2.1. Random Forest Algorithm (RF)

The random forest was proposed by Leo [20] in 2001. As the name suggests, it is a forest randomly composed of many trees. Each tree in the forest is a decision tree that can perform some judgment operations, and it belongs to the integrated learning method. When an input sample enters the random forest, each decision tree in the forest will judge and classify it and then perform scoring and evaluation to classify the sample into the category selected by the majority of decision trees. In addition, random forests are also capable of data regression and prediction. This study uses the prediction function of random forests.

**Figure 2:**
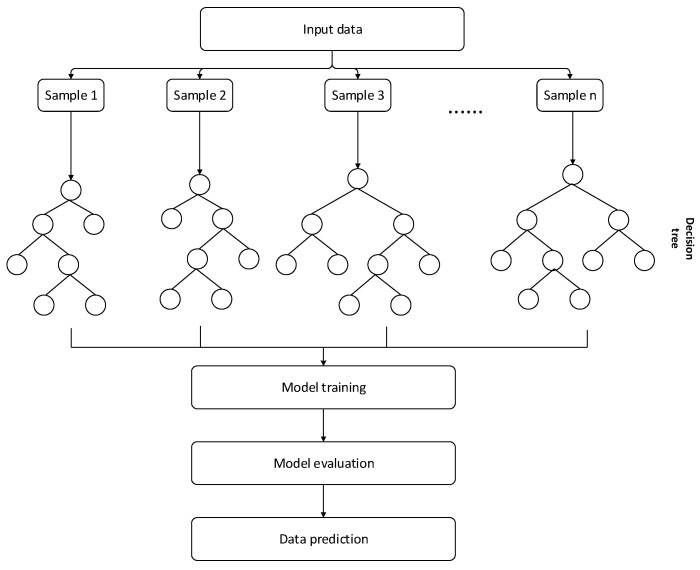
Basic framework diagram of random forest.

The steps of using a random forest for data regression and prediction include input data, decision making, model training, model evaluation, and model prediction. The random forest framework is shown in Figure 2.

It is necessary to prepare training-set and test-set data—where the training set contains known input features and corresponding output labels and the test set contains the same input features but no output labels—and ensure that all features in the data set are numerical. Next, you need to select appropriate features for training the model. In a random forest, feature importance ranking can be used to determine which features are most relevant, which can be derived by calculating the importance of each feature based on information gain or the Gini coefficient. The random forest model needs to be trained using the training-set data. A random forest contains multiple decision trees, each of which is trained using a different subset of data samples and features to avoid overfitting. The complexity and generalization ability of the random forest can be controlled by setting parameters such as the number of trees, depth, and splitting criteria. The trained model can be evaluated using test-set data, which can be measured using indicators such as the mean square error and mean absolute error. When the prediction accuracy of the model is qualified, the trained random forest model can be used to predict new input data.

Since the random forest’s data extraction process is completely random and can be repeatedly sampled with replacement, the input data of each decision tree are random, avoiding the limitations of the training model caused by the training data itself.

In the first step, according to 70% of the training samples and 30% of the test samples, the random forest is used to perform bootstrap repeated random sampling with replacement on the generated data again to generate k new sample sets $\left\{S_{k},k=1,2,\ldots \ldots ,K\right\}$.

In the second step, each sample set is input into a decision tree. At the decision node of each decision tree, *N* features are randomly selected from the *n* (*n* < *N*) features of the sample set as the basis for the classification features of the node. The total number of feature quantities remains unchanged during this decision-making process, and the principle of minimum impurity is followed. For each sample data set, after entering the decision tree, among the n features at each decision node, the feature that best matches the data is decided for branch splitting, and the decision process is repeated at the next node until the decision tree can no longer branch.

The formula for calculating impurity is as follows:

(1) $$deviance(N)=\sum_{i=1}^{N}{\left(y_{i}-\overset{-}{y}\right)}^{2}$$

In the formula, *y_i_* is the value of the *i*-th sample at the node and $\overset{-}{y}$ is the average value of all node values.

In the third step, for the selection of the final predicted value of each observation value, the random forest selects the average of the prediction results of all decision trees as the predicted value.

(2) $$H(x)=\frac{1}{k}\sum_{i=1}^{k}h_{i}(x)$$

In the formula, *H*(*x*) is the prediction result; *h_i_*(*x*) is the prediction result of each decision tree; and *k* is the number of decision trees in the forest. The prediction results are used as the input of the generative adversarial interpolation network for the next step of data filling.

### 2.2. Generative Adversarial Interpolation Network (GAIN)

The generative adversarial network (GAN) was proposed by Goodfellow et al. [21] in 2014. Its main purpose is to generate non-existent data, which allows artificial intelligence to have imagination. Generative adversarial networks are widely used in the field of image generation [22] and also in the field of data generation [23]. Using a generative adversarial network to repair and expand small sample data to a normal data size is a prerequisite for subsequent data analysis and reliability assessment.

The GAN is a deep learning model consisting of two neural networks: a generator and a discriminator. In data filling, we can use a GAN to synthesize missing data. Using existing data as a training set, a generator is trained to generate new data, which can be used to fill the gaps in the original data set.

**Figure 3:**
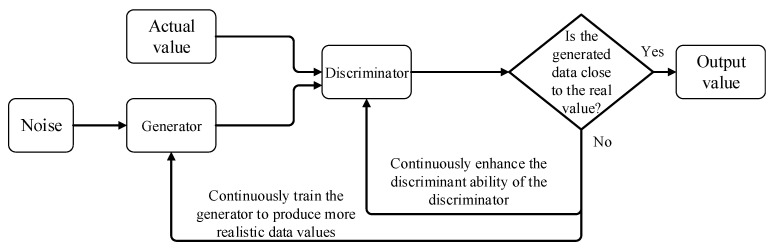
Basic framework of generative adversarial network.

During the data-filling process, the workflow of the GAN model is as follows: First, a training set needs to be prepared, which contains some known and complete data samples. Second, this training set is used to train a GAN generator model. During the training process, the generator will try to synthesize new data, and the discriminator will try its best to distinguish which data are real and which are false. Through the mutual game between the generator and the discriminator, the result generated by the generator is finally reached. The data can hardly be judged as regenerated values by the discriminator. After training is completed, the generator is used to generate new data samples, which can be used to fill the gaps in the original data set. The generative adversarial network framework is shown in Figure 3.

The optimization formula of the generative adversarial network is as follows:

(3) $$\underset{G}{\min}\underset{D}{\max}V(D,G)=E_{x-P_{data}(x)}\left[\log\left(D(x)\right)\right]+E_{z-P_{z}(z)}\left[\log\left(1-D\left(G(z)\right)\right)\right]$$

where *P_data_*(*x*) represents the distribution of the original data set, *P_z_*(*z*) represents the distribution of new samples generated by the generator, z represents the random input vector of the generator, *D*(*x*) represents the output probability of the discriminator (that is, the possibility that the input sample is a real sample), and *G*(*z*) represents the generation of the output of the processor (new sample).

Formula (3) is a problem of optimizing the maximum value and the minimum value at the same time, so the generative adversarial network adopts a rotational optimization method; that is, after optimizing the generator once, the discriminator is optimized again. When optimizing the generator, we must fix all parameters of the discriminator, take the minimum value of Formula (3), and maximize *D*(*G*(*z*)), so that the generator can generate new samples that are as realistic as possible to deceive the discriminator. When optimizing the discriminator, we must fix all parameters of the generator and take the maximum value of Formula (3), so that the discriminator can distinguish as much as possible which samples are real samples and which are false samples generated by the generator.

**Figure 4:**
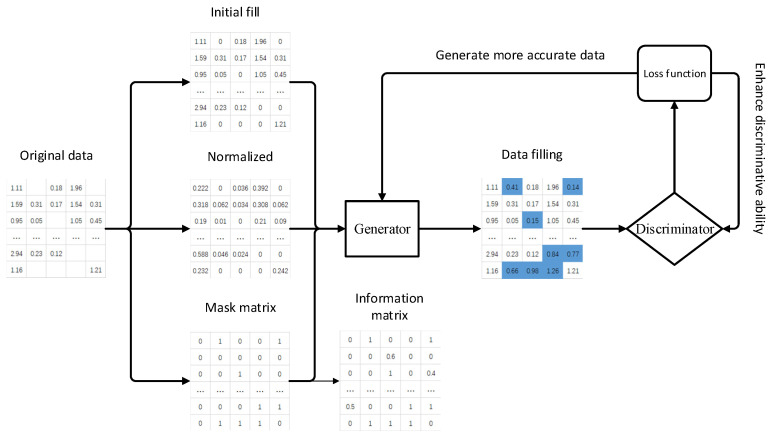
Generative adversarial interpolation network (GAIN) framework.

Generative adversarial imputation networks (GAINs) are a generalized version of GANs [24]. Their main architecture is the same as the GAN and can be used to fill in incomplete data sets. In the GAIN framework, the processing of original data is divided into three parts. First, initial filling is performed, and missing positions are filled with 0. Then, the initial filling is normalized to facilitate operations, and then a mask matrix is generated based on the original data, with the missing positions being 1; the remaining positions are 0. The generator is designed to accurately fill in missing data, and the discriminator’s goal is to distinguish whether the generator’s data are filled-in or original values. The ultimate goal of the discriminator is to minimize the classification error, while the goal of the generator is to maximize the classification error of the discriminator. Through the mutual confrontation between the two, the generated data can obtain more ideal results, and it also provides the discriminator with information about the data. The information matrix of partial information forces the samples generated by the generator to be close to the real data distribution. The generative adversarial interpolation network framework is shown in Figure 4.

### 2.3. Specific Algorithm Implementation Process

The original data set is input into the random forest algorithm, and 0 are used to replace missing positions to obtain an initial filled data set with no missing parts. We use this initial population data set to train the random forest algorithm and perform an initial population of the input data. The obtained padding data are again fed into the generative adversarial interpolation network as inputs. Through the mutual game and learning between the generator and the discriminator, an equilibrium state is reached under the adjustment of the loss function, and a filled data set close to the real distribution is obtained. The core code of the algorithm is shown in Table 1.

In Table 1, “data” represents the original data after pre-filling with 0. “filled_df” represents the data filled by the random forest. “filled_data” represents the data filled with the GAIN, and “gain_parameters” represents the gain parameter. “rmse” represents the root mean square error value.

### 2.4. Evaluation Indicators

The root mean square error (*RMSE*) is the mean square root of the square of the difference between the true value and the predicted value. The error dimension after the square root is smaller and more suitable for calculation and comparison. It is often used to evaluate the fit of a model on given data. The smaller the *RMSE*, the better the fit of the model, which means the predicted value is closer to the true value. Therefore, the research goal is to obtain the minimum value of the *RMSE*. The *RMSE* calculation formula is as follows:

(4) $$RMSE=\sqrt{\frac{1}{n}\sum_{i=1}^{n}{\left(y_{i}-y_{i}^{*}\right)}^{2}}$$

Among them, *n* is the number of data, $y_{i}$ is the real value, and $y_{i}^{*}$ is the predicted value.

The mean absolute error (*MAE*) is a method of measuring the difference between the predicted value and the actual value. It is calculated as the average of the absolute values of the difference between the predicted value and the actual value. The *MAE* calculation formula is as follows:

(5) $$MAE=\frac{1}{n}\sum_{i=1}^{n}|y_{i}-y_{i}^{*}|$$

*R*-squared is a statistic used to measure the fit of a regression model. *R*-squared ranges from 0 to 1, with values closer to 1 indicating a better fit of the model to the data. The formula for calculating *R*-squared is as follows:

(6) $$R^{2}=1-\frac{{\sum_{i=1}^{n}\left(y_{i}-y_{i}^{*}\right)}^{2}}{{\sum_{i=1}^{n}\left(y_{i}-\overset{-}{y}\right)}^{2}}$$

Among them, *n* is the number of data, $y_{i}$ is the real value, $y_{i}^{*}$ is the predicted value, and $\overset{-}{y}$ is the mean of the predicted values.

We used the *RMSE*, *MAE*, and $R^{2}$ to compare the data-filling methods. The smaller the *MAE* and *RMSE*, the larger the $R^{2}$, and the better the prediction effect. This study uses the random forest algorithm (RF), generative adversarial interpolation network (GAIN), and K-nearest neighbor classification algorithm (KNN) as the control group to explore the feasibility of the proposed missing temporal data-filling method based on a random forest and generative adversarial interpolation network (RF-GAIN).

## 3. Results and Discussion

### 3.1. Application Scenarios

For the algorithm proposed in this article, the bearing public data set of the Western Reserve University in the United States was used for a verification analysis. The experimental bench has eight normal samples, 53 bearing outer ring damage samples, 23 inner ring damage samples, and 11 rolling element damage samples. Due to the large amount of data in the public data set, this study selected the driving-end acceleration data of the base for verification. In order to better demonstrate the filling effect, the first 500 data points were selected for missing simulation and data interpolation filling.

In addition, in order to verify the effectiveness and universality of the proposed method, the algorithm was tested using the bearing anomaly data set of Western Reserve University and the operating data of the centrifugal pump test bench designed and built by ourselves.

### 3.2. Data Verification

**Figure 5:**
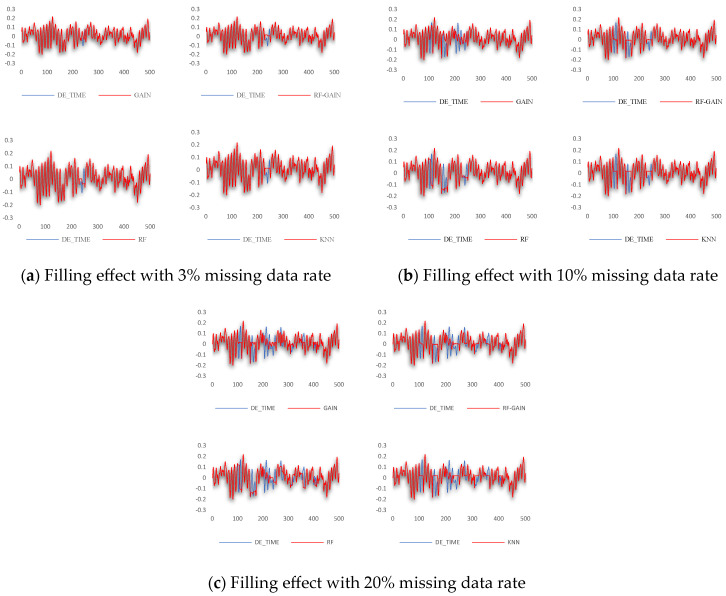
Comparison of filling effects of various algorithms under different missing data rates. (**a**) Description of filling effect with 3% missing data rate; (**b**) description of filling effect with 10% missing data rate; (**c**) description of filling effect with 20% missing data rate.

The algorithm proposed in this article was used for testing and verification in the public bearing data set of the Western Reserve University. The algorithm was used to perform data interpolation and filling when the missing data rate was 3%, 10%, and 20%, respectively. The comparison of filling effects is shown in Figure 5. Figure 5a shows the filling effect when the missing data rate is 3%. Figure 5b shows the filling effect when the missing data rate is 10%. Figure 5c shows the filling effect when the missing data rate is 20%. Each picture is shown separately in a comparison of the filling results of the GAIN algorithm, RF-GAIN algorithm, RF algorithm, and KNN algorithm with the original data results. The red part is the filling data and the blue part is the original data.

The comparison of the root mean square values of the filled data and the original data of the GAIN algorithm, RF-GAIN algorithm, RF algorithm, and KNN algorithm when the missing data rate is 3%, 10%, and 20%, respectively, is shown in Table 2.

In order to verify the applicability and robustness of the algorithm, multiple data sets are used for verification. They are the normal base data of the Western Reserve University bearing public data set (written as DATA1 in the table), the drive-end bearing rolling element failure data of the Western Reserve University bearing public data set (written as DATA2 in the table), and the centrifugal pump test designed by ourselves as data on the normal operation of the station (written as DATA3 in the table). We selected 500 pieces of data from each of the three data sets, set 10% of the data as missing, verified the data-filling effect of the algorithm, and used the RMSE, MAE, and R-squared as evaluation indicators, respectively. The specific data are shown in Table 3 below.

**Figure 6:**
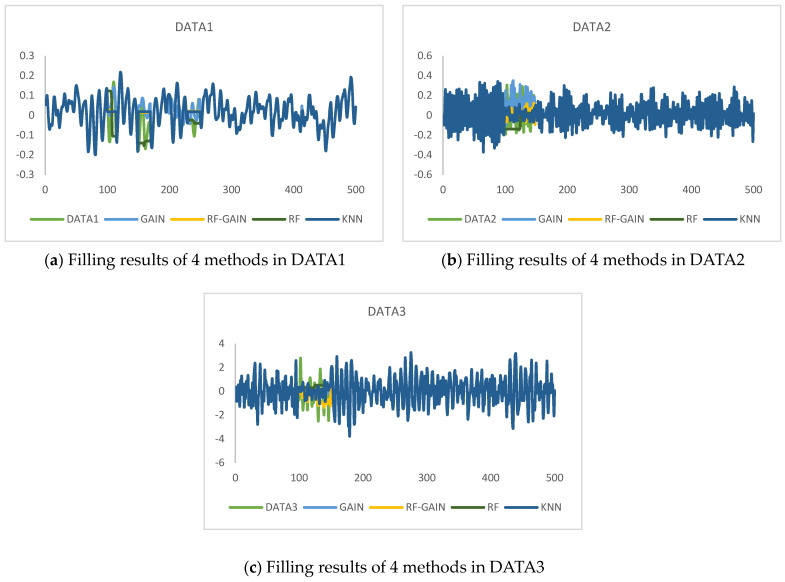
Filling performance of 4 methods on 3 data sets. (**a**) Filling results of 4 methods in DATA1; (**b**) filling results of 4 methods in DATA2; (**c**) filling results of 4 methods in DATA3.

From Table 3, the following conclusions can be initially drawn: The RF-GAIN algorithm performs better than the other three algorithms in the three data sets, which means that the algorithm has a certain universality and robustness with each data set. The filling effects of the four methods on the three different data sets are shown in Figure 6. In Discussion, the advantages and disadvantages of the RF-GAIN algorithm and the other three algorithms are discussed in detail.

## 4. Discussion

**Figure 7:**
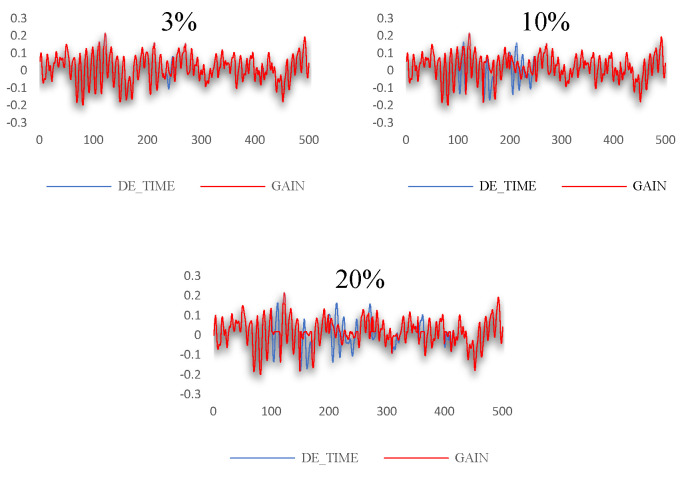
Data-filling effect of GAIN algorithm at different missing data rates.

**Figure 8:**
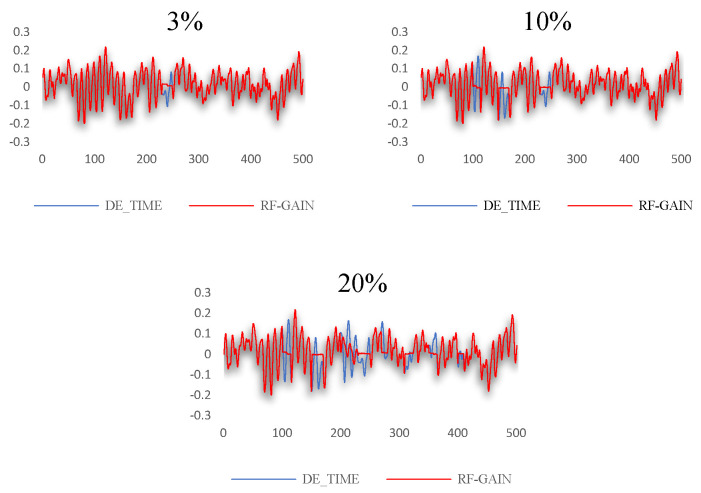
Data-filling effect of RF-GAIN algorithm at different missing data rates.

**Figure 9:**
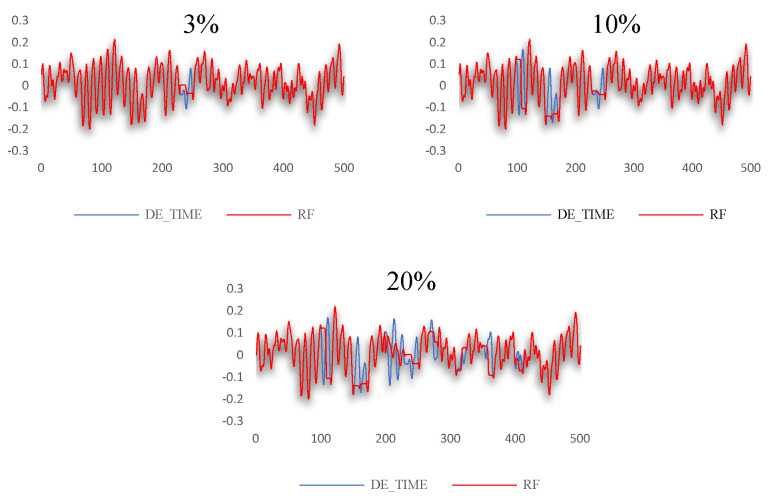
Data-filling effect of RF algorithm at different missing data rates.

**Figure 10:**
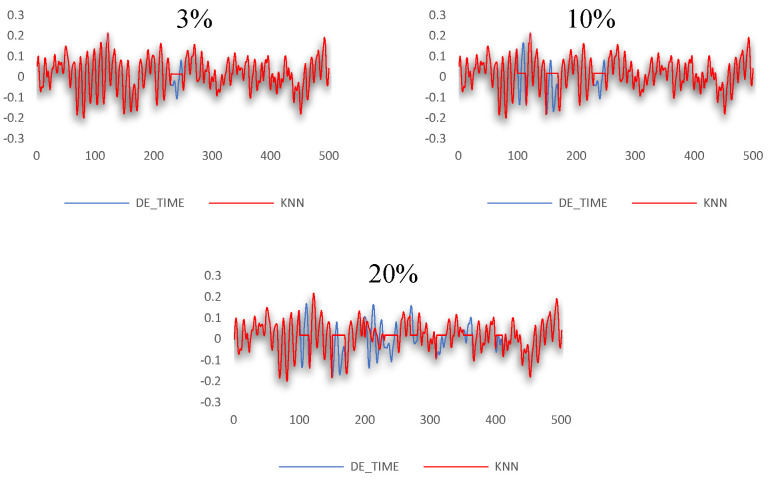
Data-filling effect of KNN algorithm at different missing data rates.

**Figure 11:**
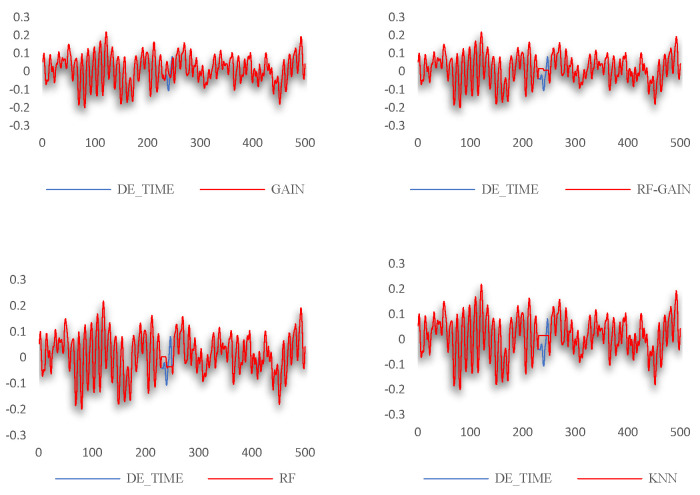
The data-filling effect of each algorithm when the missing data rate is 3%.

**Figure 12:**
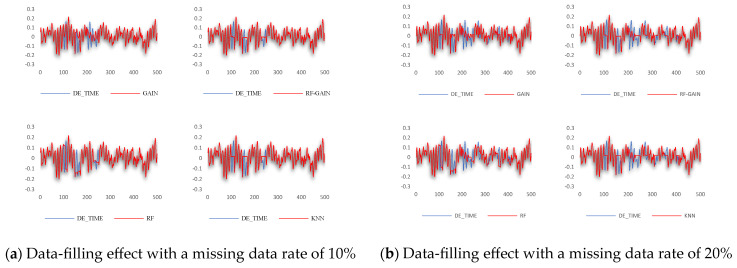
The data-filling effect of each algorithm when the missing data rate is 10% and 20%. (**a**) Description of data-filling effect with a missing data rate of 10%; (**b**) description of data-filling effect with a missing data rate of 20%.

For the data set selected in this study, the GAIN algorithm, RF-GAIN algorithm, RF algorithm, and KNN algorithm were used to conduct verification and comparative analyses when the missing data rate was 3%, 10%, and 20%, respectively. First, the filling effect of each algorithm under different missing data rates was analyzed. The analysis results are shown in Figure 7, Figure 8, Figure 9 and Figure 10. Then, the filling effects of different algorithms were analyzed under different missing data rates. The analysis results are shown in Figure 11 and Figure 12.

The GAIN algorithm performs better at data filling with different missing data rates. When the missing data rates are 3%, 10%, and 20%, the RMSE values between the filled data and the original data are 0.0162, 0.0496, and 0.0535, respectively. It can be concluded from Figure 7 that the quality of data filling decreases as the missing data rate increases. Although the data fluctuate, they have a low correlation with the change trend of the original data.

The RF-GAIN algorithm performs stably under different missing data rates. When the missing data rates are 3%, 10%, and 20%, the RMSE values between the filled data and the original data are 0.0157, 0.0386, and 0.0527, respectively. In the four comparisons, the algorithm has the lowest RMSE. It can be seen from Figure 8 that even though the data filled by this algorithm have a poor fitting degree in the change trend, the filled data fluctuate in a small range, effectively reducing the RMSE changes caused by data changes.

When the missing data rate of the RF algorithm is 3%, 10%, and 20%, the RMSE values between the filled data and the original data are 0.0168, 0.0546, and 0.0668, respectively. The data-filling effect is shown in Figure 9, ranking last among the four algorithms.

The KNN algorithm performs smoothly when processing these types of data. When the missing data rate is 3%, 10%, and 20%, the RMSE values between the filled data and the original data are 0.0173, 0.0419, and 0.0547, respectively. The trend of the fitting effects of the filled data and original data is shown in Figure 10.

In the verification of the Western Reserve University bearing data set, when the missing data rate is 3%, the root mean square values after filling by the four methods are not much different, and the values are small. The filling effect is shown in Figure 11.

The GAIN algorithm fits the missing data well in the second half, but there is a large deviation in the first half. This may be due to the fact that the structure of the generator or discriminator is not optimal and needs to be further optimized. The filling results of the RF algorithm have relatively obvious fluctuations, but they all fluctuate in the opposite direction to the original data, causing the RMSE of the results to become larger. The reason for this result may be that the learning and discriminating ability of the random forest is insufficient, and the discriminating ability of the decision tree needs to be appropriately increased. The RF-GAIN algorithm combines the above two algorithms. The RMSE of the filling result is the smallest. The filled data are near the mean of the original data and have a small change. It is the algorithm with the best filling effect. The filling effect of the KNN algorithm is the worst among the four algorithms. The filled data remain unchanged and are far from the original data. Different values of k will have an impact on the results. Therefore, the reason for the poor filling effect of the algorithm may be that the k value is not optimal. We will consider multiple verifications to find the optimal k value. Generally speaking, when the missing data rate is 3%, the RMSE of the filling results of the four algorithms is not much different. The RF-GAIN algorithm proposed in this article performs better than the other three algorithms.

When the missing data rate increases to 10% and 20%, the filling effects of the four algorithms significantly change compared to when 3% of the data are missing. The RMSE of the filling results are all above 0.03. The preliminary analysis results show multi-segment filling caused by the accumulation of errors. The filling effect is shown in Figure 12.

The GAIN algorithm can roughly simulate the data change trend when multiple segments of data are missing, but the prediction accuracy is not high, resulting in a high RMSE of the result. The filling results of the RF algorithm also have relatively obvious fluctuations, but the RMSE performance is not good. It may be that the construction of the random forest structure is not reasonable enough, resulting in large changes in the prediction results that are not in line with the actual change rules. RF-GAIN combines the above two algorithms to make the filling more conservative. The filling results fluctuate within a small range, sacrificing part of the effect of missing changes in the fitted data to ensure that the data-filling RMSE is small. This is to avoid the generalization ability of the algorithm. If it is too strong, it will lead to the excessive pursuit of trend fitting and the data will deviate far from the original value. The performance of the KNN algorithm is mediocre when multiple segments of data are missing. The generalization of the algorithm is too small. It can only fill in the data when the missing data first occurs, and keeps the filling results unchanged during this period. It requires multiple attempts with the k value to obtain the ideal results. To sum up, the RF-GAIN algorithm has better filling results.

**Figure 13:**
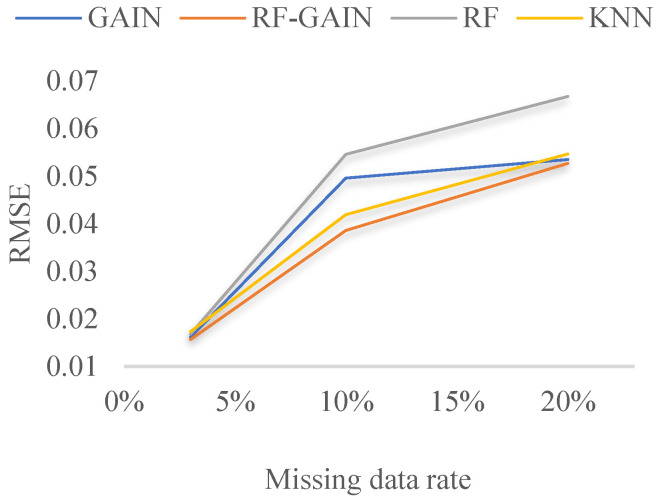
Comparison of data-filling effects of four algorithms at different missing data rates.

Overall, when the missing data rate increases, the data-filling effect of the algorithm decreases, and the RMSE value between the filled data and the original data shows an upward trend. After a comprehensive evaluation of the four algorithms, it can be seen from Figure 13 that the effect of the RF algorithm ranks last. This algorithm is sensitive to changes in the missing data rate, and the RMSE trend line of the corresponding data is separated from the other three algorithms. The filling effects of the GAIN algorithm and the KNN algorithm are in the middle. The most robust one is the RF-GAIN algorithm. In the three cases of missing data, the RMSE of the algorithm’s filled data remains at the lowest level among the four algorithms, which effectively reduces the prediction error caused by data fluctuations. However, the algorithm structure needs to be further improved to further achieve the best data trend fitting effect on the basis of maintaining the lowest RMSE. Therefore, subsequent research will focus on adjusting the RF-GAIN network structure and further optimizing the algorithm to achieve better results.

**Figure 14:**
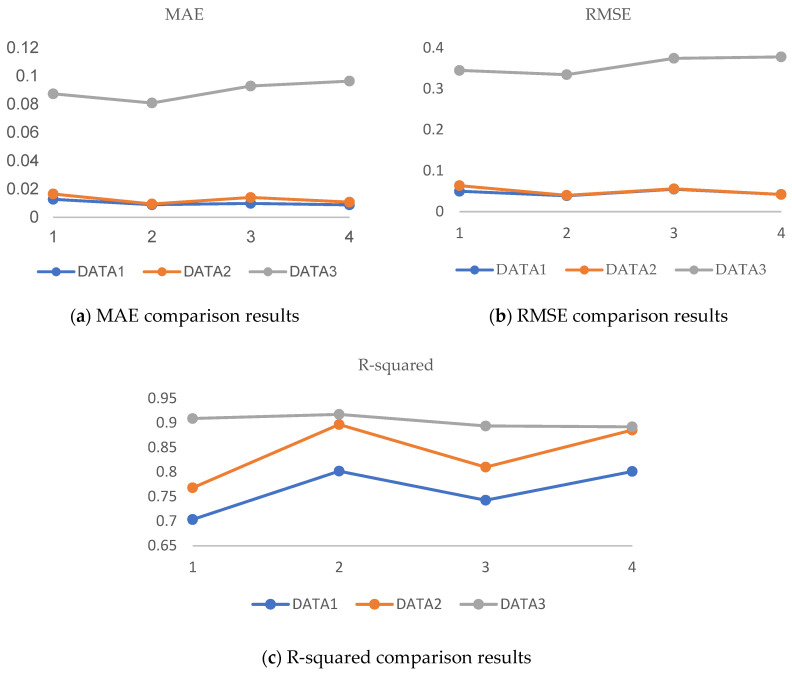
Comparison of the effects of 3 data sets.

In the analysis and comparison of multiple data sets, the filling effects of each algorithm are different. Figure 14 is a comparison of the filling effects of four algorithms in different data sets. The abscissa represents different algorithms: 1 represents the GAIN algorithm, 2 represents the RF-GAIN algorithm, 3 represents the RF algorithm, and 4 represents the KNN algorithm.

## 5. Conclusions

It is undeniable that from the above analysis of the filling results, the missing time-series data interpolation method (RF-GAIN) proposed in the article based on a random forest and generative adversarial interpolation network has better performance in data filling. In the verification of multiple data sets, the four data-filling methods have different performances. For different data types, the filling effects of each method vary. In the actual use process, there are many forms of missing data. This method can reduce the information loss caused by missing data to a certain extent and play a supporting role in subsequent data analysis. Therefore, choosing the appropriate data-filling algorithm in the appropriate application scenario can improve the data-filling effect. From the overall application effect, the RF-GAIN algorithm performs relatively well in each data set and provides a reference method in the field of data filling.

For the RF-GAIN algorithm, there is room for further optimization in future work. (1) It is necessary to optimize the random forest structure and the generator and discriminator of the generative adversarial interpolation network, as well as to enhance the adaptability and fusion of the two algorithms to be applicable to more data-filling ranges. (2) Learning and training need to be carried out on more data sets to improve the applicability of the algorithm. (3) The calculation time is long, and the algorithm structure needs to be optimized to increase the running speed.

## Figures and Tables

**Table 1:** Core code display.

Serial Number	Code	Comment
1	data.replace(0, np.nan)	Missing parts are pre-filled with 0
2	for file in csv_files	First fill of the data set using random forest
3	file_path = os.path.join(folder_path, file)	
4	data = pd.read_csv(file_path)	
5	filled_df = RandomForest (data)	
6	filled_data = GAIN(data, gain_parameters, filled_df)	Second imputation using a generative adversarial imputation network
7	rmse = rmse_loss(data, filled_data)	Calculate RMSE value
8	plt.plot(filled_data, label = ‘Filled Data’)	Display original data and filled data in one graph
9	plt.plot(data, label = ‘Original Data’)	

**Table 2:** Comparison of root mean square value of filling effect of Western Reserve University bearing data.

	Algorithm Type	GAIN	RF-GAIN	RF	KNN
Missing Data Rate					
3%		0.0162	0.0157	0.0168	0.0173
10%		0.0496	0.0386	0.0546	0.0419
20%		0.0535	0.0527	0.0668	0.0547

**Table 3:** Comparison of filling effects of 3 data sets.

	MAE				RMSE					*R* ^2^		
	GAIN	RF-GAIN	RF	KNN	GAIN	RF-GAIN	RF	KNN	GAIN	RF-GAIN	RF	KNN
DATA1	0.0127	0.0089	0.0098	0.0088	0.0496	0.0386	0.0546	0.0419	0.7032	0.8016	0.7422	0.8008
DATA2	0.0165	0.0094	0.0140	0.0107	0.0633	0.0397	0.0556	0.0418	0.7677	0.8965	0.8097	0.8851
DATA3	0.0874	0.0809	0.0929	0.0964	0.3448	0.3343	0.3742	0.3779	0.9087	0.9172	0.8935	0.8919

## Data Availability

The data presented in this study are available on request from the corresponding author.
